# A Novel TGR5 Activator WB403 Promotes GLP-1 Secretion and Preserves Pancreatic β-Cells in Type 2 Diabetic Mice

**DOI:** 10.1371/journal.pone.0134051

**Published:** 2015-07-24

**Authors:** Chunbing Zheng, Wenbo Zhou, Tongtong Wang, Panpan You, Yongliang Zhao, Yiqing Yang, Xin Wang, Jian Luo, Yihua Chen, Mingyao Liu, Huaqing Chen

**Affiliations:** 1 Shanghai Key Laboratory of Regulatory Biology, Institute of Biomedical Sciences and School of Life Sciences, East China Normal University, Shanghai, China; 2 Institute of Biosciences and Technology, Department of Molecular and Cellular Medicine, Texas A&M University Health Science Center, Houston, Texas, United States of America; Broad Institute of Harvard and MIT, UNITED STATES

## Abstract

The G protein-coupled receptor TGR5 is a membrane receptor for bile acids. Its agonism increases energy expenditure and controls blood glucose through secretion of glucagon-like peptide-1 in enteroendocrine cells. In this study, we explored the therapeutic potential of WB403, a small compound activating TGR5 which was identified by combining TGR5 targeted luciferase assay and active GLP-1 assay, in treating type 2 diabetes. After confirmation of TGR5 and GLP-1 stimulating activities in various cell systems, WB403 was examined in oral glucose tolerance test, and tested on different mouse models of type 2 diabetes for glycemic control and pancreatic β-cell protection effect. As a result, WB403 exhibited a moderate TGR5 activation effect while promoting GLP-1 secretion efficiently. Interestingly, gallbladder filling effect, which was reported for some known TGR5 agonists, was not detected in this novel compound. *In vivo* results showed that WB403 significantly improved glucose tolerance and decreased fasting blood glucose, postprandial blood glucose and HbA1c in type 2 diabetic mice. Further analysis revealed that WB403 increased pancreatic β-cells and restored the normal distribution pattern of α-cell and β-cell in islets. These findings demonstrated that TGR5 activator WB403 effectively promoted GLP-1 release, improved hyperglycemia and preserved the mass and function of pancreatic β-cells, whereas it did not show a significant side effect on gallbladder. It may represent a promising approach for future type 2 diabetes mellitus drug development.

## Introduction

Diabetes, with its complications, has long become a global public health problem in the twenty-first century [1, 2]. Type 2 diabetes mellitus (T2DM) is the most common form of diabetes characterized mainly by impaired function of pancreatic β-cells or peripheral insulin resistance [3, 4]. Currently, many diabetic drugs are available on market, such as insulin, biguanides insulin sensitizer metformin, sulfonylureas insulin secretagogue glibenclamide, thiazolidinediones (TZDs) peroxisome proliferator-activated receptor gamma (PPAR-γ) agonist pioglitazone, glucagon-like peptide-1 (GLP-1) receptor agonist exenatide, dipeptidyl peptidase-4 (DPP-4) inhibitor sitagliptin, etc. However, many of these medications have various side effects. For example, metformin has been associated with gastrointestinal irritation. Glibenclamide is a major cause of drug-induced hypoglycemia. Pioglitazone has been withdrawn in some countries because of high risk of bladder cancer. Other side effects include weight gain and increased possibility of accelerating function loss of pancreatic β-cells [5–7]. Another serious problem is, despite aggressive treatment, glycemic control may still deteriorate. As a result, new therapeutic agents that could better improve glycemic control with less adverse effects are urgently needed.

Recent years, the gut hormone GLP-1 targeted therapies such as GLP-1 mimetics and DPP-4 inhibitors have been widely used in treating type 2 diabetes. Data showed that GLP-1 stimulates β-cell differentiation, survival, proliferation, and has the potency to stimulate insulin secretion in a glucose-dependent manner [8–10]. DPP-4 inhibitors or GLP-1 receptor agonists could control glycemia in diabetic mice by extending or mimicking GLP-1 function respectively [11–13]. However, these two therapies did not increase secretion of endogenous GLP-1, so that they may be unable to halt the progression of the disease because lacking of some local effects that endogenous secreted GLP-1 might have [14]. It is anticipated that therapies directly targeting intestinal L cells to stimulate GLP-1 secretion will have certain advantages [14, 15]. T2DM patients may retain some GLP-1 secretion ability which should be considered in the long-term treatment. G protein coupled receptors (GPCRs) GPR119, GPR120, GPR40 and TGR5 are predominantly expressed in intestine, where they were found on enteroendocrine L-cells, which make these receptors exciting targets for the development of therapeutic L cell secretagogues [16–18]. An increasing number of studies focusing on small compounds targeting these receptors were reported, showing significant improvement in hyperglycemic control by stimulating GLP-1 secretion in diabetic rodent models and cell systems [17, 19–21]. Among various models, *db/db* mice and the nongenetic HFD/STZ mice model of type 2 diabetes has been widely used to mimic human type 2 diabetes.

TGR5, a membrane receptor for bile acids (BAs) [22], is highly expressed in intestine, brown adipose tissue and liver [23, 24]. It was demonstrated that TGR5 activation was related to enhanced energy expenditure and attenuated obesity [24]. More importantly, TGR5 signaling pathway is crucial in regulating GLP-1 secretion. Therefore, TGR5 has been recognized as a promising incretin-based strategy for the treatment of diabetes [17]. Data showed that TGR5 stimulation by BAs induced GLP-1 production [25]. Even the relatively weak BA, ursodeoxycholic acid (UDCA), has been reported to increase GLP-1 secretion in human subjects via TGR5 signaling [26], thus had been put on phase IV clinical trial in combination with sitagliptin.

In an attempt to develop a TGR5 mediated GLP-1 secretagogue, a small compound library was established mainly based on reported TGR5 agonists [27, 28]. By combining target-based and phenotypic screening, we identified one compound, WB403, which stimulated TGR5 activity moderately, while promoted GLP-1 secretion potently. Furthermore, it did not cause gallbladder filling which is a common problem reported for TGR5 agonists. Further investigation demonstrated that WB403 effectively reduced fasting blood glucose (FBG) and postprandial blood glucose (PBG) as well as the glycosylated hemoglobin A1c (HbA1c) of T2DM mice, which is associated with preservation of β-cell mass and function. In conclusion, WB403 may have the potential to be developed in the future as anti-diabetic therapeutics.

## Materials and Methods

### Ethics statement

All animal procedures were reviewed and approved by the Animal Ethics Committee of East China Normal University (Permit number: AR2013/07001). Animal experiments were performed in strict accordance with the guidelines for laboratory animal care and use, and all efforts were made to minimize suffering of the animals. The SPF animal facility in East China Normal University has the license for small animal experiments (SYXK (Hu) 2010–0094), given by the Science and Technology Commission of Shanghai Municipality, China. A completed ARRIVE guidelines checklist is included in S1 Checklist.

### Animals and treatment

Five-week old male ICR mice and *db/db* (C57BL BKS cg-M+/+ lepr-/-) mice were purchased from Shanghai SLAC Laboratory Animal Co., Ltd. (Shanghai, China). Mice were housed 4–5 per cage and allowed access to normal chow diet and autoclaved water freely. They were fed at a constant room temperature (25°C) with a 12-h light (7:00 AM)/dark (7:00 PM) cycle. All the mice were monitored at least daily, and no adverse events were observed. In general, the *db/db* mice show a high food intake at 4-week old and become obese and develop type 2 diabetes at 8-week age. Postprandial 2 h blood glucose was assessed and mice with the similar degree of hyperglycemia and body weight were randomized into different groups in order to get a similar average PBG in each group.

For generation of nongenetic mice model of type 2 diabetes, ICR mice were fed with high fat diet (HFD, with 60% fat, Research Diets) for three weeks, then fasted for 5 h before receiving a single dose of streptozotocin (STZ, Sigma) intraperitoneally (ip) at 80 mg/kg to induce partial insulin deficiency. After three more weeks’ HFD feeding, majority of HFD/STZ-treated mice displayed hyperglycemia. Diabetes was assessed using conventional criteria [11, 29]. Mice with similar degree of hyperglycemia and body weight were randomized into various groups in order to get a similar average PBG in each group.

Diabetic mice in group of four were treated by gavage with vehicle (0.5% carboxymethylcellulose, CMC, Sigma), WB403 50, 100 mg/kg or sitagliptin at 100 mg/kg daily for 4 weeks in *db/db* mice (n = 5 with chow diet), and 8 weeks in HFD/STZ mice (n = 8 with HFD, additional normal mice group with chow diet). During the entire dosing period, body weight, food intake and water intake were monitored three times per week, while FBG and PBG were measured weekly. Overnight fasted mice received an oral glucose challenge at 2 g/kg and postprandial 2 h blood glucose was measured. Animals were treated and assessed in the order of the vehicle, WB403 50, 100 mg/kg and sitagliptin 100 mg/kg group.

### Compounds

WB403, 4-(5-((4-bromobenzyloxy)-methyl)-4-methylthiazol-2-yl)-2-ethylpyridine was synthesized in our lab. GPR119 agonist GSK1292263 was purchased from Selleck. GPR40 agonist linoleic acid was purchased from Sigma and TAK875 from Biochempartner. DPP-4 inhibitor sitagliptin phosphate was obtained from Darui, Shanghai, China.

### Cell culture

Human colon cancer cell line NCI-H716 and 293T cell line was purchased from the Cell Bank of Chinese Academy of Sciences in Shanghai. Mouse MIN6 cells, a pancreatic β-cell line established by Miyazaki et al. [30], were donated by Prof. Xiaoying Li (Shanghai Ruijin Hospital, Shanghai, China). NCI-H716 cells were grown in RPMI-1640 medium supplemented with 10% fetal bovine serum (FBS) (Gibco) at 37°C in a humidified atmosphere with 5% CO2 and 95% air, and other cells were grown in high glucose DMEM medium (Hyclone).

### GPCR analyses

For human TGR5 luciferase (luc) assay, 293T cells were co-transfected with pCRE-luc, pTGR5 (kind gift from Prof. Johan Auwerx, Ecole Polytechnique Fédérale de Lausanne, Lausanne, Switzerland) and pRenilla (Promega, USA). WB403 was added 5 h later. Cells were harvested after 16 h and subjected to luciferase analysis on Lumistar Optima chemiluminescent detector (BMG labtech, Germany). For cAMP testing, 293T cells transiently transfected with pTGR5 or pGPR119 (GeneCopoeia, USA) were washed by FBS-free DMEM, and stimulated with WB403 in FBS-free DMEM containing 3-isobutyl-1-methyl-xanthine (IBMX, Sigma). Cell lysates were collected 30 min later and used for cAMP ELISA assay. FLIPR Calcium 5 assay kit (Molecular Devices, USA) was used for calcium mobilization assay. 293-GPR40 stable cells (kindly provided by Prof. Naiming Zhou of Zhejiang University, Hanzhou, China) [31] or 293 cells transient transfected with GPR120 were seeded at 50,000 cells per well in black-walled clear-bottomed 96-well plates (Costar). An equal volume of loading buffer containing Fluo 4-AM was added the following day and cells were incubated for 1 h before stimulated with compounds. Increase of the intracellular Ca^2+^ concentration was monitored by Flexstation 3 system (Molecular Devices, USA).

### GLP-1 secretion

Endocrine differentiation was induced by seeding NCI-H716 cells in plates coated with Matrigel (Becton Dickinson). Cells were washed with RPMI-1640 medium without FBS and incubated in the same medium containing indicated compounds at 10 μmol/l. The condition medium was collected after 30 min. GLP-1 secretion in MIN6 or primary enterocytes was conducted similarly. To isolate primary enterocytes, ileum or colon was cut into 2 cm length, rinsed by PBS (pH7.2∼7.4) before dispersed in 10 mmol/l EDTA/PBS (pH7.4) for 15 min at room temperature, followed by pipetting up and down. Enterocytes were collected by filtration through a 70 μm cell strainer. To study the effect of WB403 on GLP-1 secretion in vivo, mice in group of three were fasted overnight and orally received 0, 50, 100 mg/kg of WB403 dispersed in 0.5% CMC with a DPP-4 inhibitor (5 mg/kg). There were 5 animals in each group. 2 g/kg glucose was administrated 30 min later and blood was collected 10 min after glucose challenge.

### Enzyme-linked immunosorbent assay (ELISA)

The GLP-1 (active) ELISA kit was purchased from Millipore, and insulin ELISA kit was from Mercodia. For quantitative determination of cAMP, a competitive enzyme immunoassay kit from R&D system was used. All immunoassays were performed according to manufacturers’ instructions.

### Oral glucose tolerance test (OGTT)

Mice were fasted overnight (14 h) before glucose tolerance tests. Normal mice received a 4 g/kg glucose challenge and for diabetic mice it’s 2 g/kg. WB403 at 50, 100 mg/kg, or sitagliptin at 100 mg/kg were administrated orally 30 min prior to glucose load (n = 10) [19, 32]. For vehicle group, 0.5% CMC was administrated. Blood glucose levels were measured from tail bleeds with a glucometer (Roche, Accu-Chek Performa) at specified time points. Animals were treated and assessed in the order of the vehicle, WB403 50, 100 mg/kg and sitagliptin 100 mg/kg group.

### Pharmacokinetic test

Adult male Sprague-Dawley rats fasted for 12 h were randomized into two groups (n = 5) for intravenous injection (i.v.) or oral administration (p.o.). Blood samples were collected from the orbital plexus at 10, 20, 30, 40, 60, 90, 120, 150, 180, 240, 360 and 480 min after administration. The concentration of WB403 in plasma was determined by an Aligent Technologies 1260 series HPLC system with a DAD detector. The oral bioavailability (F%) was calculated as F (%) = AUC_0-∞_(p.o.) / AUC_0-∞_(i.v.) × Dose (i.v.) / Dose (p.o.).

### Histological analyses

Analysis of islet cell mass was performed using conventional methods [11, 33]. In brief, mice were sacrificed by cervical dislocation. The body part of the pancreas was rapidly excised, fixed in 10% neutral buffered formalin solution for 24 h and embedded in paraffin, then sectioned at 5 μm ready for hematoxylin and eosin (H&E) staining and immunohistochemistry staining (IHC). The latter was carried out using rabbit anti-insulin polyclonal antibody (Cat. no. 4590, 1:600) and rabbit anti-glucagon monoclonal antibody (Cat. no. 8233s, 1:200) from Cell Signaling Technology.

### Statistical analysis

Data were presented as mean ± SEM unless otherwise stated. Statistical significance between two groups was evaluated by two-tailed Student’s unpaired t-test. For multiple groups, one-way ANOVA was used. p<0.05 was considered statistically significant.

## Results

### A novel small compound WB403 activating TGR5 stimulated GLP-1 secretion

**Fig 1:**
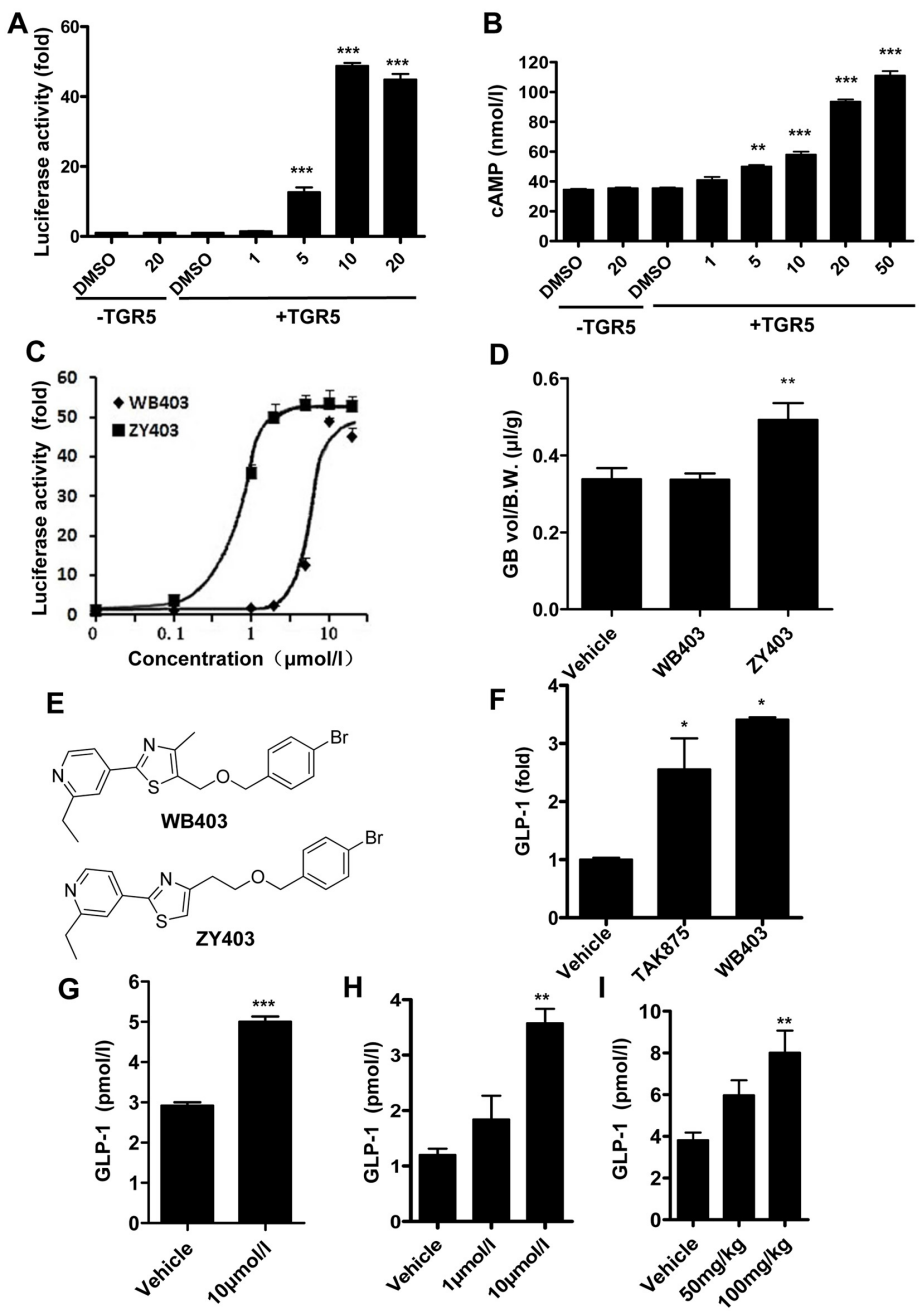
WB403 activated TGR5 and promoted active GLP-1 secretion. (A) WB403 stimulated hTGR5 in CRE-luciferase report system at the concentration range of 1–20 μmol/l. (B) WB403 stimulated hTGR5 specific cAMP accumulation at the concentration range of 1–50 μmol/l. n = 3. **p<0.01, ***p<0.001 vs. DMSO (+TGR5) group. (C) The hTGR5 targeted CRE-luciferase activity of WB403 and ZY403. EC_50_ was 5.5 μmol/l and 1.3 μmol/l for WB403 and ZY403 respectively. (D) Effect of WB403 and ZY403 on gallbladders of mice. Normal mice were fasted overnight and treated with compounds (200 mg/kg) or vehicle (DMSO) by ip injection. Gallbladders (GB) were removed 30 min later and volumes measured then normalized to body weight (B.W.). n = 10. **p<0.01 vs. vehicle group. (E) Structure of WB403 and ZY403. WB403 promoted GLP-1 secretion in NCI-H716 cells (F), primary enterocytes (G), and MIN6 cells (H). n = 3. *p<0.05, **p<0.01, ***p<0.001 vs. vehicle group. (I) WB403 promoted GLP-1 in ICR mice. n = 5. **p<0.01 vs. vehicle group. Values represent mean ± SEM.

A total of 100 compounds were screened through combination of TGR5 targeted luciferase assay and active GLP-1 immunoassay (S1 Fig). Through primary luciferase screen, 11 compounds were identified using the criteria of more than 10 fold increase as compared to DMSO control. These compounds were subsequently tested on GLP-1 assay. As shown by the results, TGR5 luciferase activity and GLP-1 activity did not always align with each other: the one that had the highest luciferase activity (ZY403) did not show a strong stimulation for GLP-1 activity, on the other hand, WB403 with relatively lower luciferase activity, showed much higher GLP-1 stimulation capacity (S1 Fig). Subsequent assessment confirmed that WB403 dose-dependently stimulate luciferase reaction as well as Gs-coupled cAMP accumulation through TGR5 mediated pathway (Fig 1A and 1B). The efficiency of stimulation was lower by WB403 than by ZY403 (Fig 1C), with a TGR5 EC_50_ of 5.5 μM for WB403, similar to those for BAs [17, 34]. The responses were receptor-mediated as no stimulation of luciferase or cAMP activity was shown in control 293T cells transfected with an empty plasmid. When effects of these two compounds on gallbladder were tested, it was shown that ZY403 at 200 mg/kg caused gallbladder filling in mice, but WB403 at the same dose did not (Fig 1D).

Further experiments showed that WB403 had an outstanding effect in GLP-1 stimulation in NCI-H716 cells as well as primary enterocytes (Fig 1E–1G). In addition, the GLP-1 stimulation effect of WB403 was confirmed in mouse MIN6 cells (Fig 1H). MIN6 cells also produce GLP-1 although they are widely used as a pancreatic β-cell-specific cell line [35, 36]. Moreover, *in vivo* results showed that oral treatment of WB403 significantly increased GLP-1 concentration in serum (Fig 1I). Altogether, WB403, which has a moderate stimulating activity to TGR5, demonstrated potent stimulation for GLP-1 secretion while showed no side effect of gallbladder filling.

### WB403 improved OGTT in normal and diabetic mice

**Fig 2:**
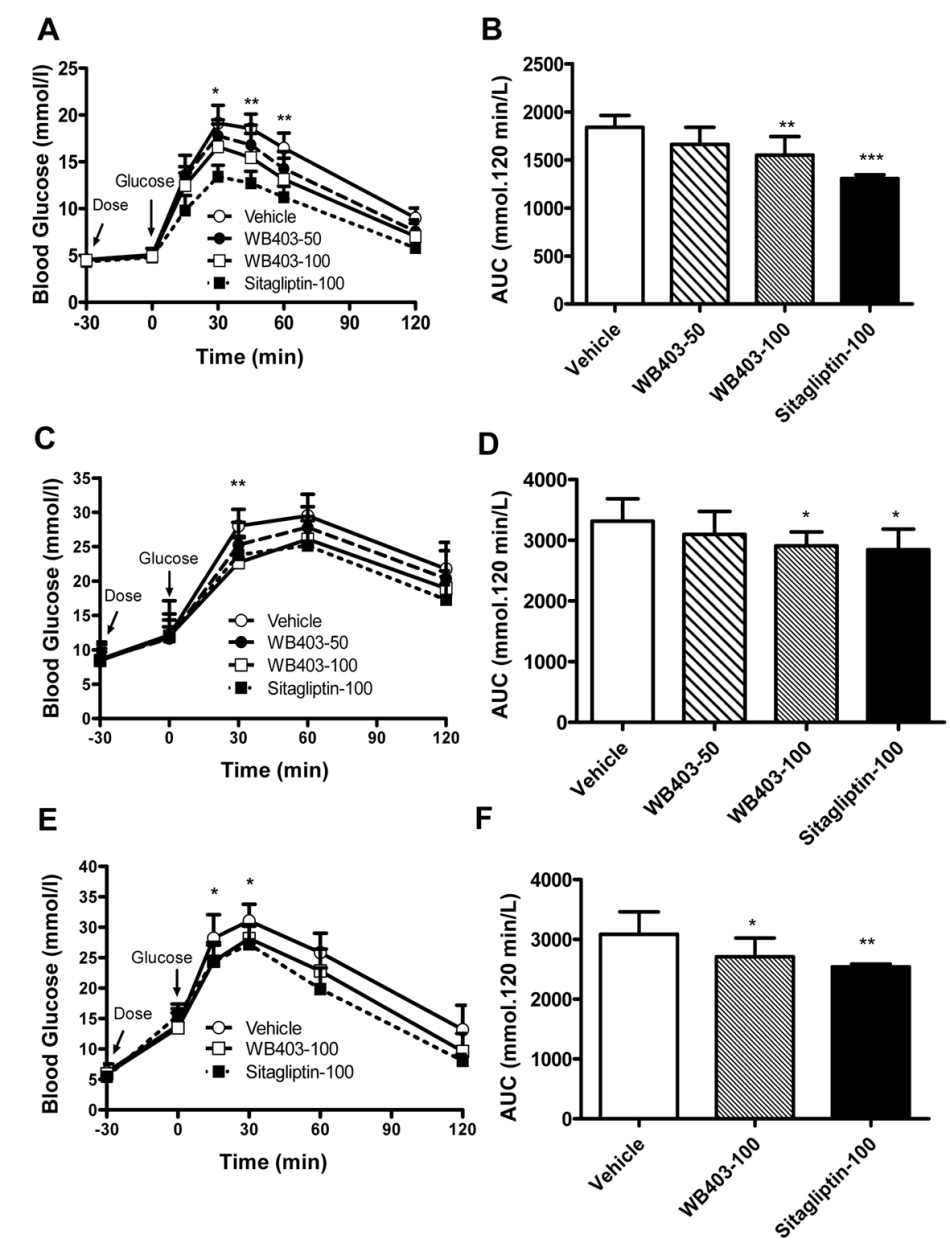
Effects of WB403 on glucose tolerance during OGTT in normal and diabetic mice. OGTT time course and AUC in normal mice (A, B), HFD/STZ mice (C, D) and *db/db* mice (E, F). Values represent mean ± SD (n = 10). *p<0.05, **p<0.01, ***p<0.001 vs. vehicle group. Asterisks in A, C and E reflect significance between WB403 100 mg/kg and vehicle group.

Since WB403 significantly promoted GLP-1 secretion, the effect of WB403 on blood glucose undulation was evaluated. It was found that in normal mice, WB403 administration caused a significant improvement in blood glucose tolerance compared with vehicle group (Fig 2A and 2B). The same results were exhibited both in HFD/STZ mice (Fig 2C and 2D) and *db/db* mice (Fig 2E and 2F).

WB403 at 20 μmol/l did not show significant toxicity on cell viability in 293T, MIN6 and NCI-H716 cells after 24 h exposure (S2 Fig). Additionally, there was no obvious toxicity manifested in ICR mice subjected to a 7-day’s acute toxicity test when receiving a single dose of WB403 with a concentration gradient from 2000 to 6000 mg/kg, although stress was observed in mice at 6000 mg/kg dosage. Sections of tissues in WB403-treated mice did not show obvious signs of toxicity (S2 Fig). Furthermore, pharmacokinetic test showed that oral bioavailability of WB403 was 12.0%, and the elimination half-life (T_1/2_) value after oral administration of 40 mg/kg WB403 was 176.4 ± 64.6 min (Table 1).

**Table 1:** The pharmacokinetic parameters of WB403 in Sprague-Dawley rats.

Parameters	p.o. (40 mg/kg)	i.v. (40 mg/kg)
T_1/2_ (min)	176.4 ± 64.6	166.0 ± 25.8
T_max_ (min)	84.0 ± 6.7	-
C_max_ (μg/mL)	0.98 ± 0.16	-
C_0_ (μg/mL)	-	25.7 ± 4.6
AUC_0-t_ (min*μg/mL)	136.7 ± 21.4	1499.4 ± 150.3
AUC_0-∞_ (min*μg/mL)	193.1 ± 36.8	1612.7 ± 184.6
Bioavailability	12.0%	-

The oral bioavailability (F%) was calculated as F (%) = AUC_0-∞_(p.o.) / AUC_0-∞_(i.v.) × Dose (i.v.) / Dose (p.o.). Values are mean ± SEM (n = 5).

### Effects of WB403 in treatment of type 2 diabetic mice

**Fig 3:**
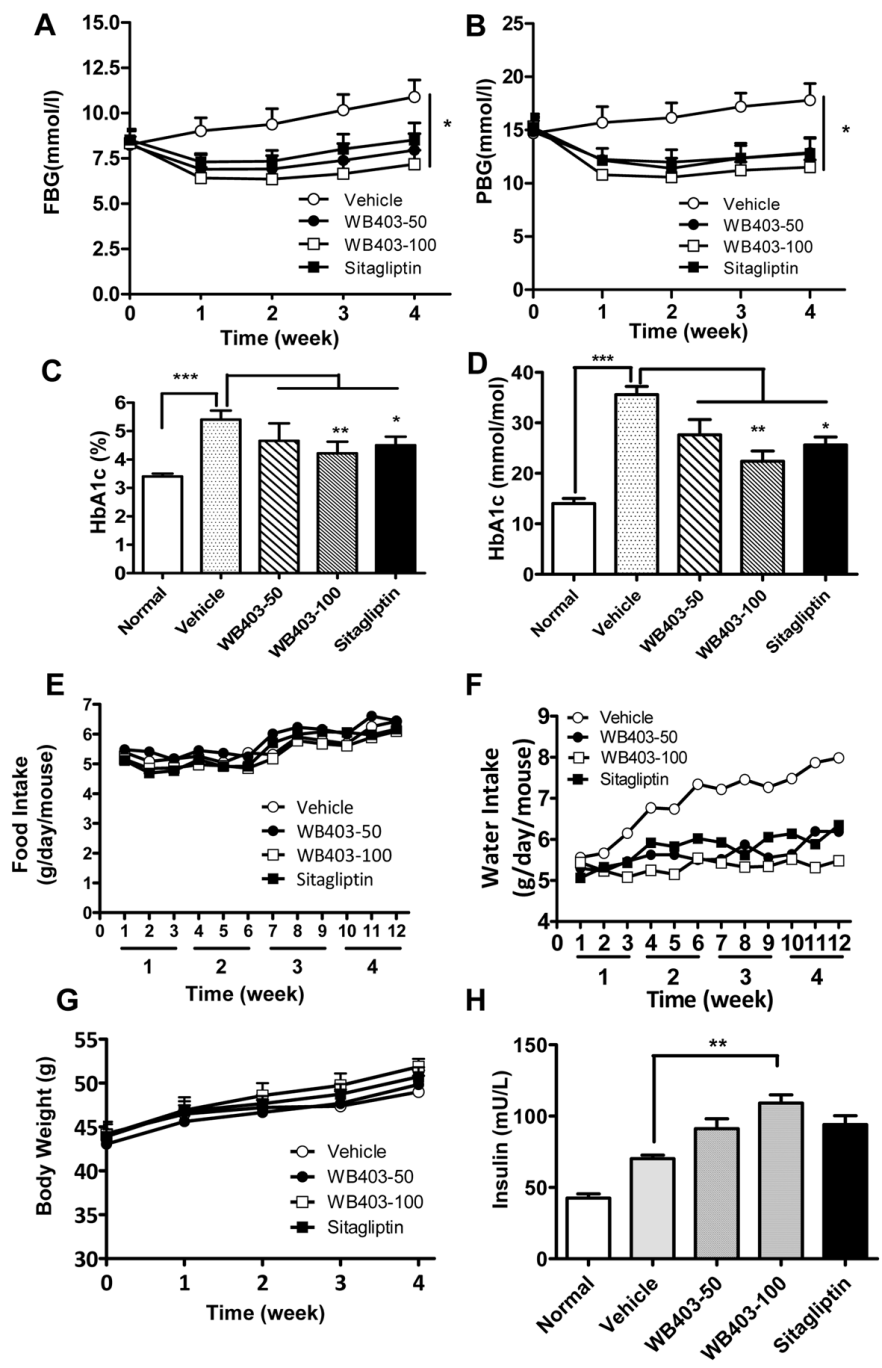
Effects of WB403 treatment in *db/db* mice. (A, B) FBG and PBG measured every week during the 4-week treatment period. (C, D) Serum levels of HbA1c, measured at the end of intervention by Adicon Clinical Laboratories (Shanghai, China) using an immunoturbidimetric assay on Beckman AU680 biochemical analyzer. (E) Average daily food intake per mouse during the treatment period. Data was collected three times per week. (F) Average daily water intake per mouse. (G) Body weight from different groups of mice. (H) Serum levels of insulin were measured at the end of intervention. Values are mean ± SEM (n = 5). *p<0.05, **p<0.01, ***p<0.001 vs. vehicle group.

Next we proceeded to investigate the effect of WB403 in treating T2DM. *db/db* mice were administrated with WB403 at the time when diabetes features emerged. After one week of WB403 intervention, mice showed a significantly decreased FBG and PBG (Fig 3A and 3B) as compared to vehicle group. More importantly, HbA1c levels in WB403-treated groups were significantly lower as compared to the vehicle group (Fig 3C and 3D). While food intake and body weight did not vary significantly, water intake was decreased in WB403-treatment groups, indicating an improvement in hyperglycemia (Fig 3E–3G). Additionally, there were higher insulin levels in treatment groups (Fig 3H).

**Fig 4:**
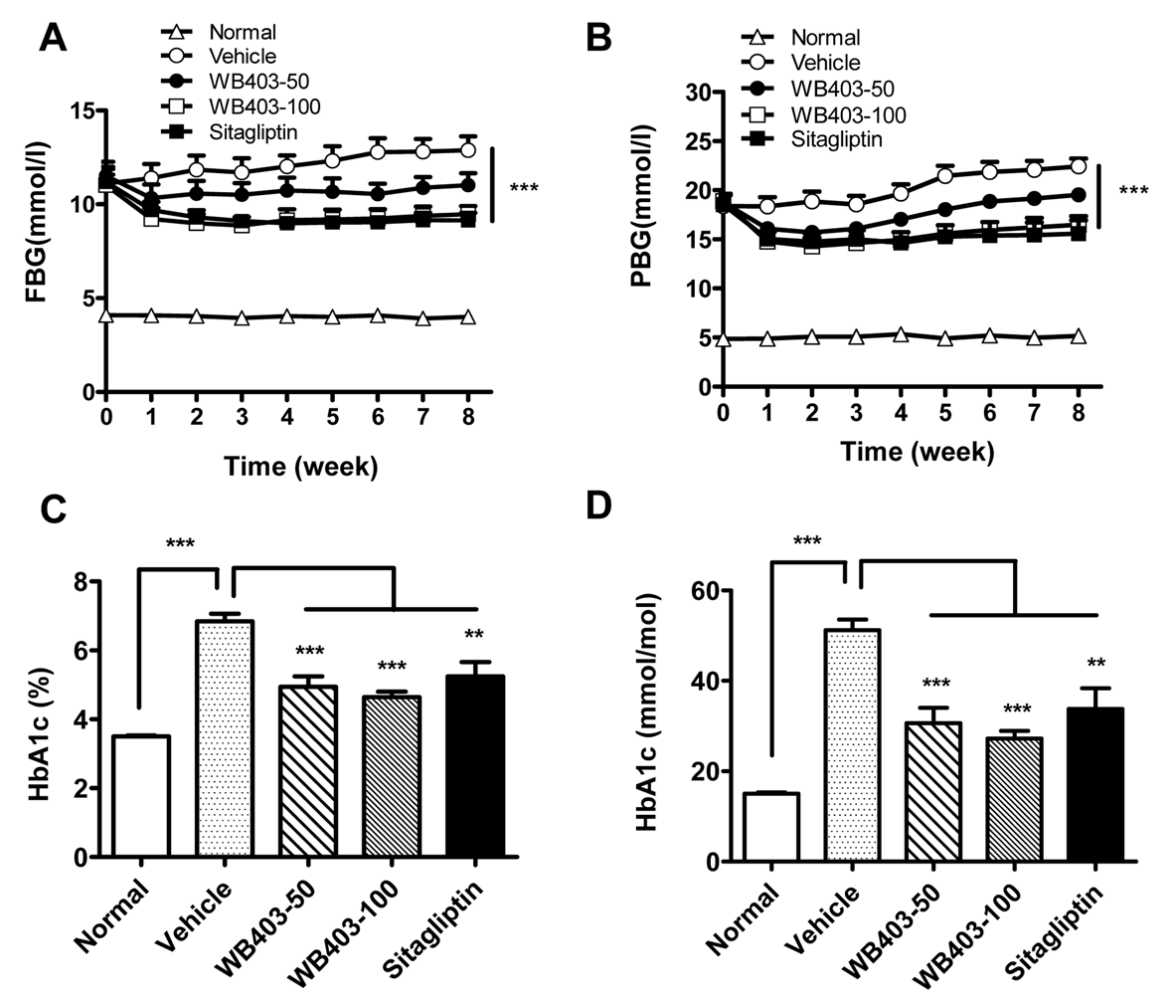
WB403 improved hyperglycemia of HFD/STZ mice. Mice were treated with different concentration of WB403, vehicle or sitagliptin. FBG (A) and PBG (B) were measured every week during the 8-week treatment period. (C, D) HbA1c levels in serum. Values are mean ± SEM (n = 8). *p<0.05, **p<0.01, ***p<0.001 vs. vehicle group.

The effect of WB403 in treating diabetes was further demonstrated in HFD/STZ mice. As shown in Fig 4, administration of WB403 significantly decreased FBG and PBG in HFD/STZ mice, starting from the first week’s intervention and extending throughout the whole treatment period. This finding suggested that WB403 had a long standing effect. Consistent with the result in *db/db* mice, serum HbA1c levels from WB403-treated HFD/STZ mice were decreased significantly as compared to that in vehicle control mice (Fig 4C and 4D). In addition, WB403 treatment in normal mice did not cause significant FBG and PBG lowering (S3 Fig). Thus, WB403 treatment decreased FBG, PBG and HbA1c in type 2 diabetic mice, but did not show obvious hypoglycemia risk in normal mice.

### WB403 preserved the mass and function of pancreatic β-cells

**Fig 5:**
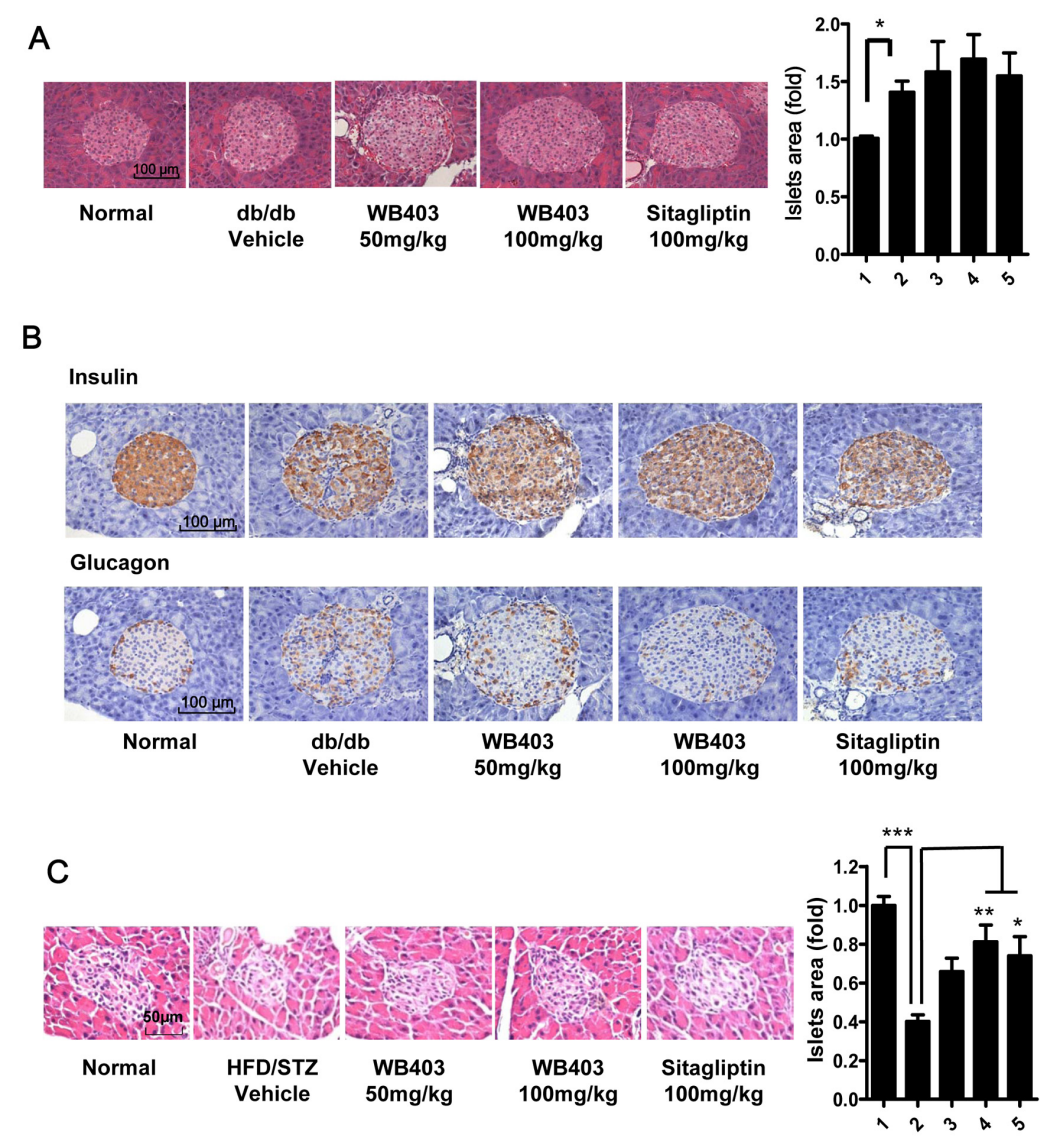
WB403 preserved the mass of pancreatic β-cells and normal distribution of α and β-cells. (A) H&E staining of pancreas from *db/db* mice, and statistical result. Islets were sized by the Image J analysis software on alternated pancreatic sections spaced each by 100 μm. (B) Immunohistochemical analysis of pancreatic sections by anti-insulin antibody or anti-glucagon antibody. (C) H&E staining of pancreas from HFD/STZ mice, and statistical result. Results are representative islets from each group. 1–5 in the column graph on the right represents normal mice, diabetic mice treated with vehicle, WB403 50 mg/kg, WB403 100 mg/kg, sitagliptin 100 mg/kg respectively (n = 5). *p<0.05, **p<0.01, ***p<0.001 vs. diabetes-vehicle group.

The above experiments showed that WB403 had good sustained glycemic control efficiency and increased insulin secretion. We further investigated whether these effects were related to pancreatic β-cells. Islet sections from vehicle and WB403-treated *db/db* mice were analyzed (Fig 5A). Vehicle *db/db* mice displayed a bigger islet area as compared to normal mice because of obesity and insulin resistance. Mice from WB403 or sitagliptin-treatment group showed a bigger islet area as compared to the vehicle mice, though no significant difference was found. When we examined the distribution of α-cell and β-cell in islets, we found islets of normal mice comprised of a large insulin positive β-cell core surrounded by a small quantity of glucagon positive α-cells. In contrast, islets from *db/db* mice contained a lot more glucagon positive cells, which infiltrated the entire islet including the core area. Sections from WB403 and sitagliptin groups showed significantly reduced α-cell in the islet core area and restored the normal cell distribution pattern (Fig 5B and S4 Fig). Different from *db/db* mice, HFD/STZ mice showed a smaller islet area contrast to normal mice because of β-cell lose induced by STZ. Treatment with WB403 restored islet area but did not expand it beyond that of normal mice (Fig 5C). Above results demonstrated that WB403-treatment in diabetic mice had a good β-cell protective effect.

### Effects of WB403 on other GPCRs related to GLP-1 secretion

**Fig 6:**
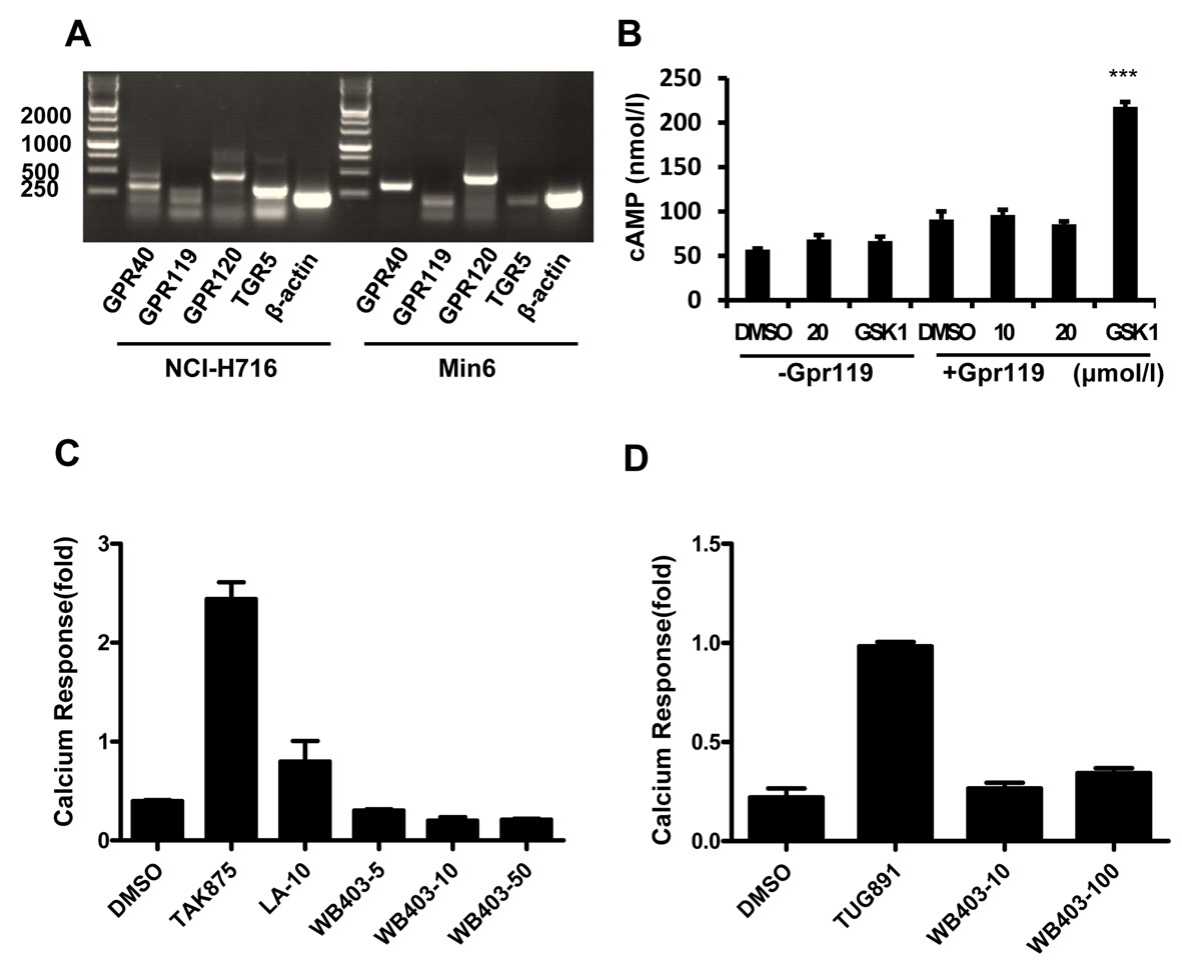
Effect of WB403 on GPCRs related to GLP-1 secretion. (A) GPCR expression in human NCI-H716 and mouse MIN6 cells. In NCI-H716 cells, the amplified fragment is 457bp, 190bp, 452bp, and 290bp for hGPR40, hGPR119, hGPR120, hTGR5 respectively. In MIN6 cells, the amplified fragment is 332bp, 190bp, 470bp, and 190bp for mGpr40, mGpr119, mGpr120, mTgr5 respectively. (B) WB403 at 10, 20 μmol/l had no significant stimulation effect on hGPR119 dependent cAMP accumulation. (C) WB403 at 5, 10, 50 μmol/l had no significant effect on hGPR40-Ca^2+^ activation. (D) WB403 at 10 or 100 μmol/l had no significant effect on hGPR120-Ca^2+^ activation. GSK1292263 (1 μmol/l), TAK875 (0.1 μmol/l) and linoleic acid (LA, 10 μmol/l), TUG891 (10 μmol/l) were used as positive agonist of GPR119, GPR40 and GPR120 respectively. Data are representative of three experiments.

In addition of TGR5, agonists against other GPCRs such as GPR40, GPR119 and GPR120 have been reported to stimulate GLP-1 secretion. As it seems possible that TGR5 activation capacity of WB403 might only represent part of its GLP-1 stimulation effect, we examined whether WB403 could also activate other GPCRs. Results showed that GPR40, GPR119 and GPR120 were expressed in NCI-H716 and MIN6 cells (Fig 6A). However, WB403 did not show significant stimulation activity to GPR119 dependent cAMP accumulation (Fig 6B), neither did it exhibit significant effect on GPR40 or GPR120 as manifested by GPR40 or GPR120-dependent calcium mobilization (Fig 6C–6D and S5 Fig). Therefore, it was concluded that WB403 did not have an effect on GLP-1 stimulating GPCRs such as GPR40, GPR119 and GPR120.

## Discussion

In this study, a novel compound WB403 was identified, which stimulated GLP-1 activity through TGR5 pathway. Unique features about WB403 included its moderate TGR5 activation capacity (similar to BAs [17, 34]), and potent GLP-1 stimulation activity. Interestingly, the commonly reported side effect of gallbladder filling associated with known TGR5 agonists was not detected in WB403, making it an attractive candidate for potential drug development that may have a beneficial advantage in anti-diabetic therapy.

Since TGR5 was recognized as a promising therapeutic target for type 2 diabetes treatment, efforts has been put in identifying and characterizing various TGR5 agonists. Despite the potentially optimistic therapeutic prospects, problems with side effects of TGR5 agonists were frequently reported, such as gallstone formation and gallbladder filling [37, 38]. To eliminate such toxic side effects of TGR5 agonists, a much lower systemic exposure or even a non-systemic exposure was suggested [39]. In our study, although the mechanism is not completely clear, the finding that no side effect of gallbladder filling was detected in WB403-treated mice was very encouraging. Whether the absence of gallbladder filling was related to the fact that WB403 only moderately regulated TGR5 yet potently stimulate GLP-1 activity, or simply because WB403 may has a lower systemic exposure is yet to be determined. WB403 exhibited a moderate activity on TGR5 (EC50 at 5.5 μM), similar to those of moderate bile acids such as chenodeoxycholic acid (CDCA, EC50 at 4.43 μM), cholicacid (CA, EC50 at 7.72 μM) [22, 34]. Most TGR5 agonists reported to produce gallbladder filling have better TGR5 activity: lithocholic acid (LCA) 0.58 μM; INT-777 0.82 μM [17, 34, 38]. On the other hand, pharmacokinetic parameters suggested that elimination time of WB403 in plasma was not very long, and its concentration in plasma was relatively low, which implied that although not restricted in the colon area, only a small portion of the oral administered WB403 reached the systemic circulation, so that the most important effect of WB403 was restricted in the intestine. As TGR5 is distributed in the intestine and GLP-1 is secreted by intestinal L-cells, WB403 may mainly exert its action through stimulating GLP-1 secretion in intestine. Hence by oral administration, WB403 could provide an adequate activity in intestine, avoiding systemic toxicity to other parts of the body.

WB403 treatment in vivo showed very good effects on both FBG and PBG which are usually taken as indications for function of pancreas β-cells in clinical cases [40–42]. Furthermore, WB403 also exhibited excellent suppression effect on HbA1c. In clinical research, even slight reduction of HbA1c level is correlated with significant decreases of T2DM complications which are always responsible for decreased life quality [43, 44]. These findings suggested a good therapeutic action of WB403 on diabetes and impaired β-cells. As T2DM progresses, blood glucose level rises but β-cell function declines. So, the preservation effect of WB403 on pancreas β-cells is very meaningful. Histology analysis showed that treatment of WB403 in diabetic mice elevated β-cell mass in islets. WB403 may exert its effect on β-cells through increasing islet cell proliferation, or through inhibition of apoptosis. The function on pancreas β-cells is reflected not only in the mass and insulin secretion ability but also in the distribution of β-cell and α-cell [11, 45]. Administration of WB403 restored the cell distribution pattern in islets and therefore protected the β-cell function. It is known that GLP-1 and GLP-1 mimetics have potential to improve pancreatic function through promotion of islets survival and proliferation. Our result indicated that WB403 also exerted a sustainable effect on glycemic control via improved β-cell status.

The TGR5 specificity in luciferase and cAMP assay suggested that the effect of WB403 was at least in part mediated through TGR5 pathway. Furthermore, part of WB403’s effect in animal might attribute to its effect on energy expenditure in addition to its GLP-1 stimulating capacity [24]. It is reasonable to predict that administration of WB403 in combination with sitagliptin could give additive beneficial effects in T2DM therapy. The fact that WB403 had a moderate stimulation activity to TGR5 but a high GLP-1 promotion capacity suggested that there might be other targets in function. To this end, we tried to analyze whether WB403 could stimulate other GPCRs related to GLP-1 secretion. The initial ones tested in our study included GPR40, GPR119 and GPR120 [39]. In contrary to our hypothesis, data showed that WB403 did not have significant effect on any of the GPCRs tested. Whether the activity of WB403 is related to some other GLP-1 stimulating receptors or even multiple targets requires further study.

In conclusion, the newly identified bioavailable GLP-1 secretagogue WB403, with its *in vitro* and *in vivo* data showing TGR5 modulation and GLP-1 activation capacity, glucose tolerance improvement, preservation for mass and function of pancreatic β-cells, as well as absence of gallbladder filling side effect, represented a promising novel candidate for anti-diabetic drug development. The identification and characterization of WB403 not only add a new member to the growing list of TGR5 activators, generating possibilities for development of new drugs that can better control diabetes while overcoming unfavorable side effects, but also shed lights to the mechanism of disease control strategy that involves GPCRs and GLP-1. Further studies in elucidating the mechanism of regulation and action of WB403 will be beneficial.

## Supporting Information

S1 Checklist Completed “The ARRIVE Guidelines Checklist” for reporting animal data in this manuscript. (PDF)

S1 Fig Screening of small compounds by CRE-luc assay and active GLP-1 assay. Compounds were screened by TGR5 CRE-luc assay on 293T cells (A), and the selected compounds were further analyzed for GLP-1 secretion on NCI-H716 cells (B). Fold of luc increase was labeled on the corresponding column of selected compounds.
(TIF)

S2 Fig Toxicity analyses of WB403. Toxicity analyses of WB403. (A) WB403 at 20 μmol/l did not show significant toxicity on cell viability after 24 h exposure. Cell viability was determined by MTS assay. Values are mean ± SD (n = 3), *p<0.05, **p<0.01 vs. control group. (B) H&E staining of tissue sections from vehicle group and 6000mg/kg WB403 group ICR mice after a 7-day’s acute toxicity test.
(TIF)

S3 Fig WB403 treatment in normal mice did not cause significant FBG and PBG lowering. Mice were treated for 4 weeks by WB403 and sitagliptin at 100 mg/kg. Glibenclamide at 2.5 mg/kg was used as positive control for hypoglycemia. FBG (A) and PBG (B) were measured every week. Values are mean ± SD (n = 10). *p<0.05, **p<0.01 vs. vehicle group.
(TIF)

S4 Fig WB403 preserved the mass of pancreatic β-cells. Immunofluorescence staining of pancreatic sections from *db/db* mice by anti-insulin antibody. Results are representative islets from each group.
(TIF)

S5 Fig Representative original data for GPR40 and GPR120 dependent calcium mobilization assay. (A) In 293-GPR40 stable cells, WB403 at the concentration range of 5–50 μmol/l did not exhibit significant effect on calcium mobilization. (B) In 293 cells transient transfected with human GPR120 expression vector, WB403 at the concentration range of 10–100 μmol/l did not exhibit significant effect on calcium mobilization.
(TIF)
