# Supplementary figures and images for: A Novel TGR5 Activator WB403 Promotes GLP-1 Secretion and Preserves Pancreatic β-Cells in Type 2 Diabetic Mice
Source: PLoS One. 2015 Jul 24;10(7):e0134051. doi: 10.1371/journal.pone.0134051 (PMC4514850; doi:10.1371/journal.pone.0134051)

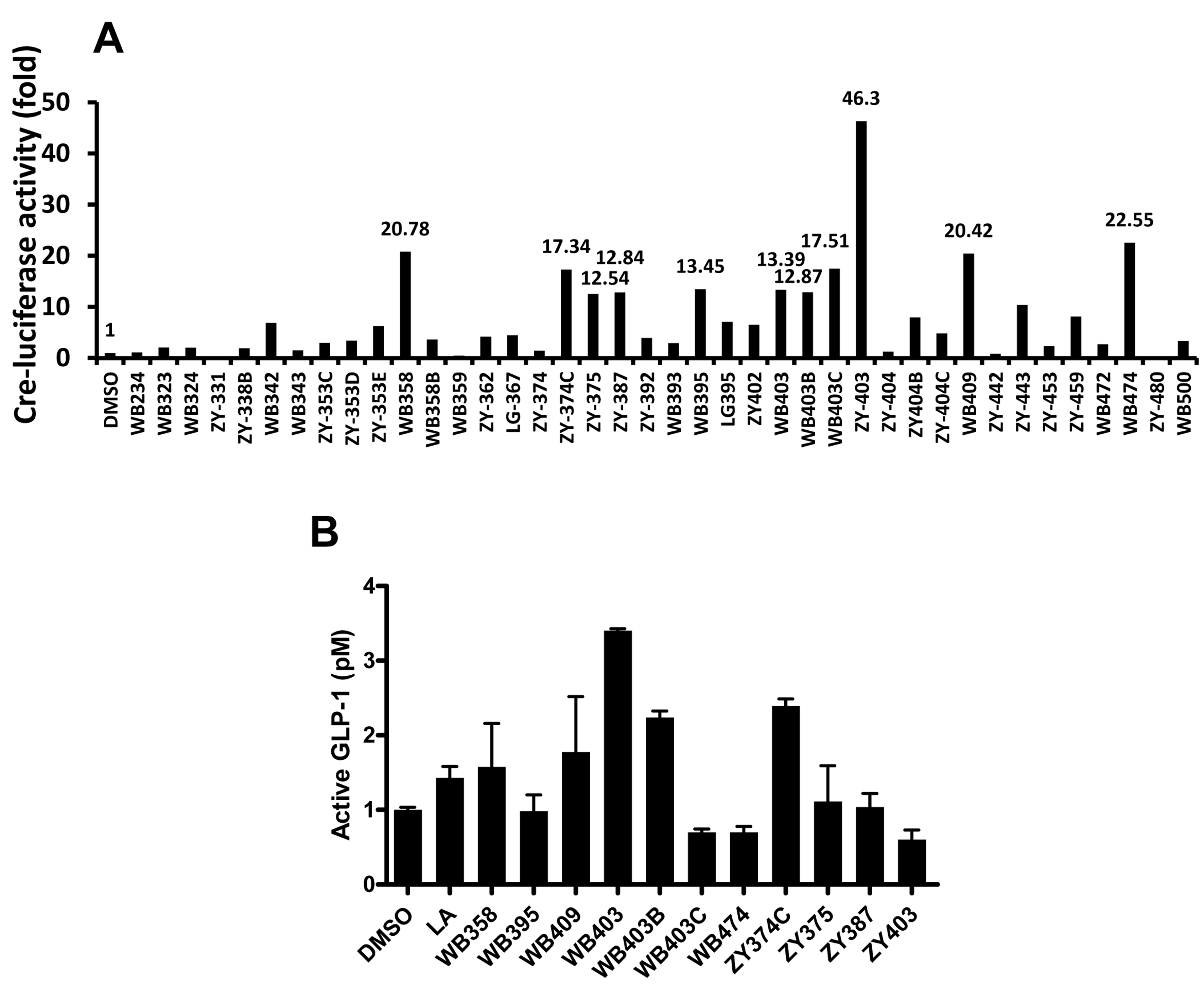

Supplement: S1 Fig — Compounds were screened by TGR5 CRE-luc assay on 293T cells (A), and the selected compounds were further analyzed for GLP-1 secretion on NCI-H716 cells (B). Fold of luc increase was labeled on the corresponding column of selected compounds. (TIF) [file pone.0134051.s002.tif]

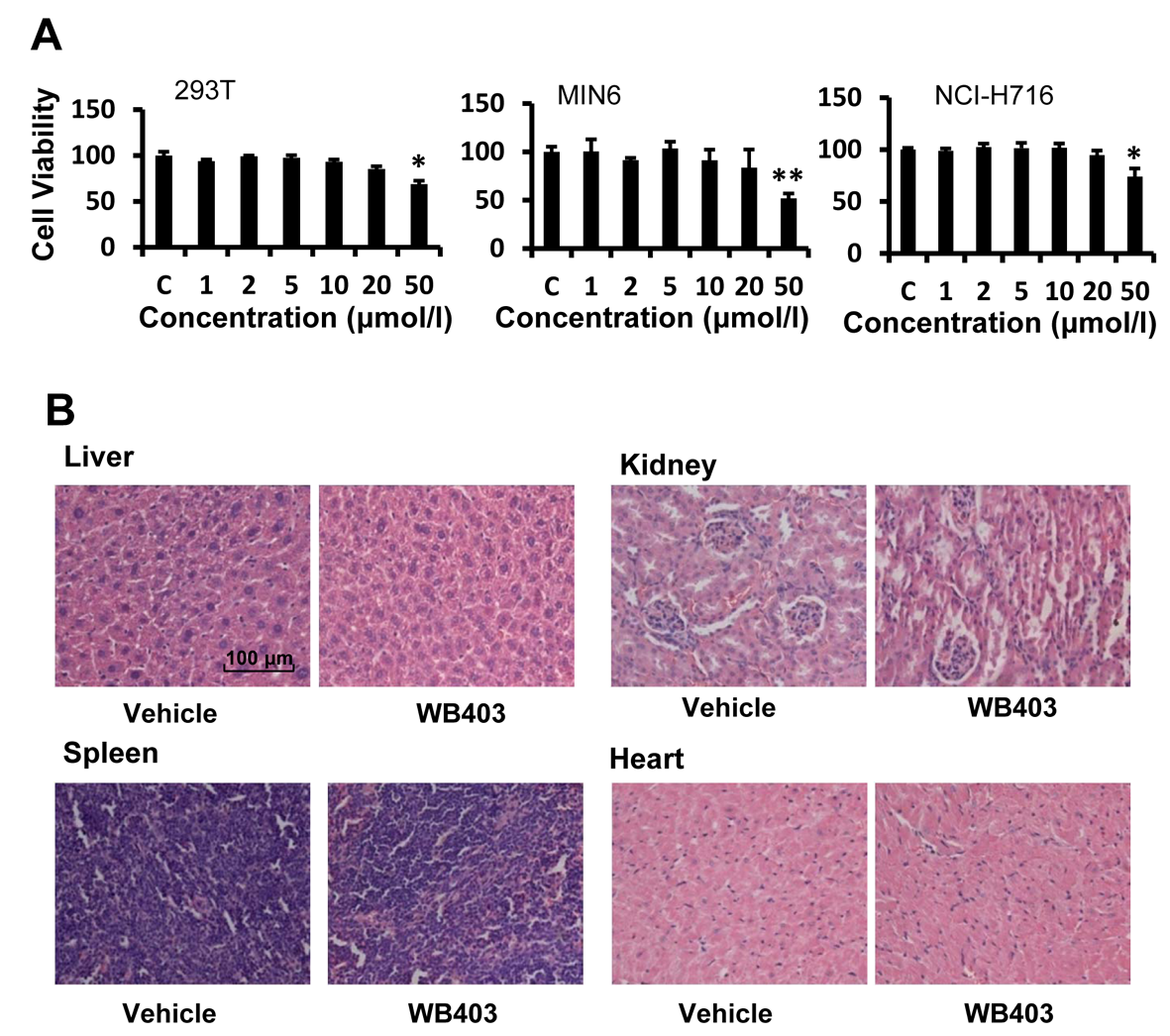

Supplement: S2 Fig — Toxicity analyses of WB403. (A) WB403 at 20 μmol/l did not show significant toxicity on cell viability after 24 h exposure. Cell viability was determined by MTS assay. Values are mean ± SD (n = 3), *p<0.05, **p<0.01 vs. control group. (B) H&E staining of tissue sections from vehicle group and 6000mg/kg WB403 group ICR mice after a 7-day’s acute toxicity test. (TIF) [file pone.0134051.s003.tif]

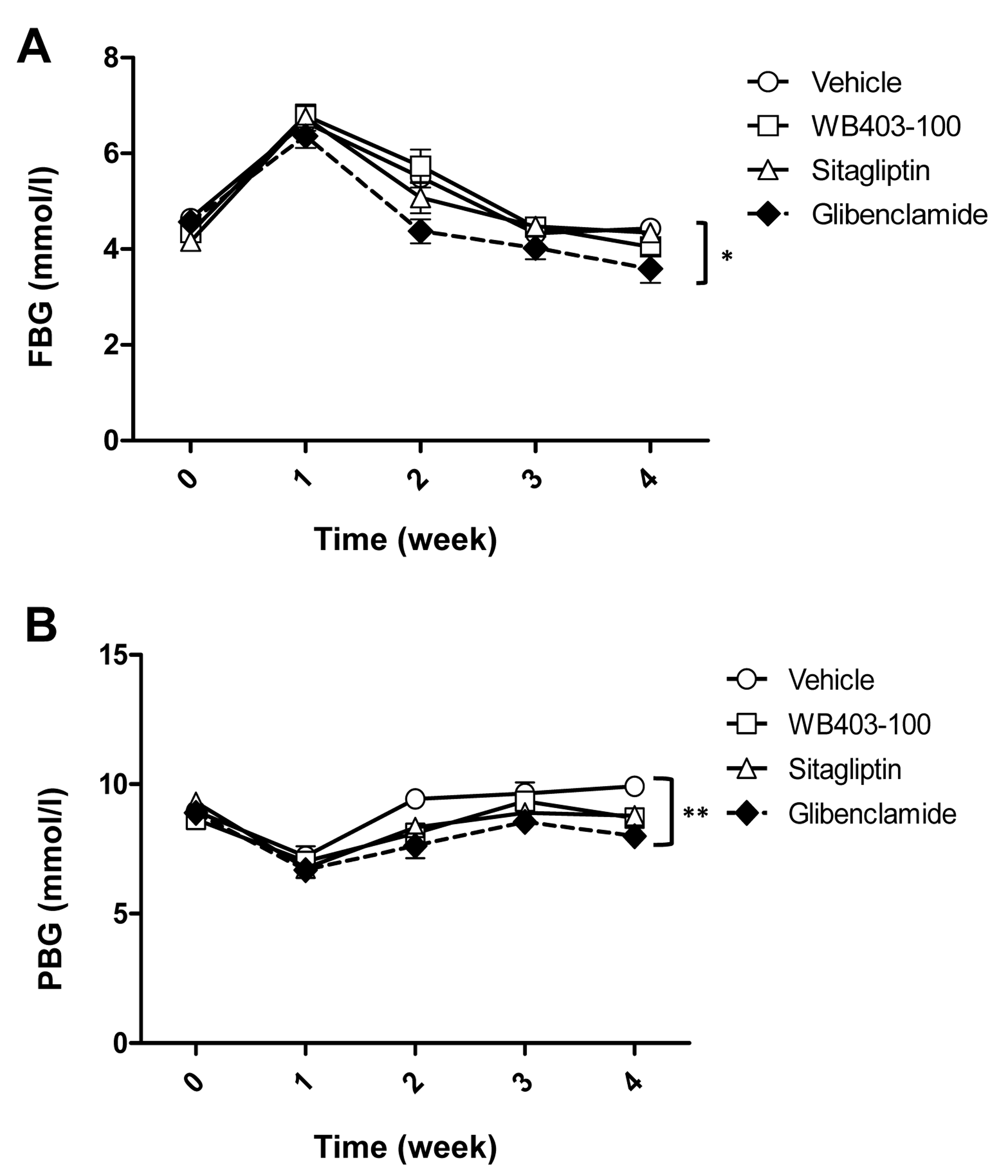

Supplement: S3 Fig — Mice were treated for 4 weeks by WB403 and sitagliptin at 100 mg/kg. Glibenclamide at 2.5 mg/kg was used as positive control for hypoglycemia. FBG (A) and PBG (B) were measured every week. Values are mean ± SD (n = 10). *p<0.05, **p<0.01 vs. vehicle group. (TIF) [file pone.0134051.s004.tif]

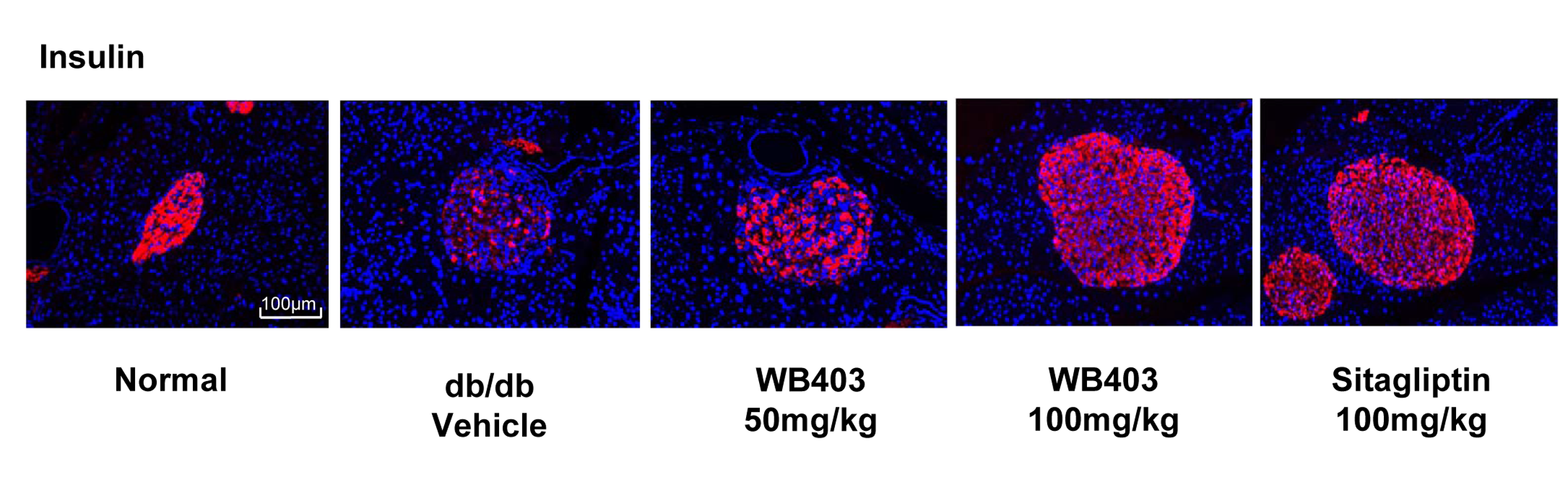

Supplement: S4 Fig — Immunofluorescence staining of pancreatic sections from db/db mice by anti-insulin antibody. Results are representative islets from each group. (TIF) [file pone.0134051.s005.tif]

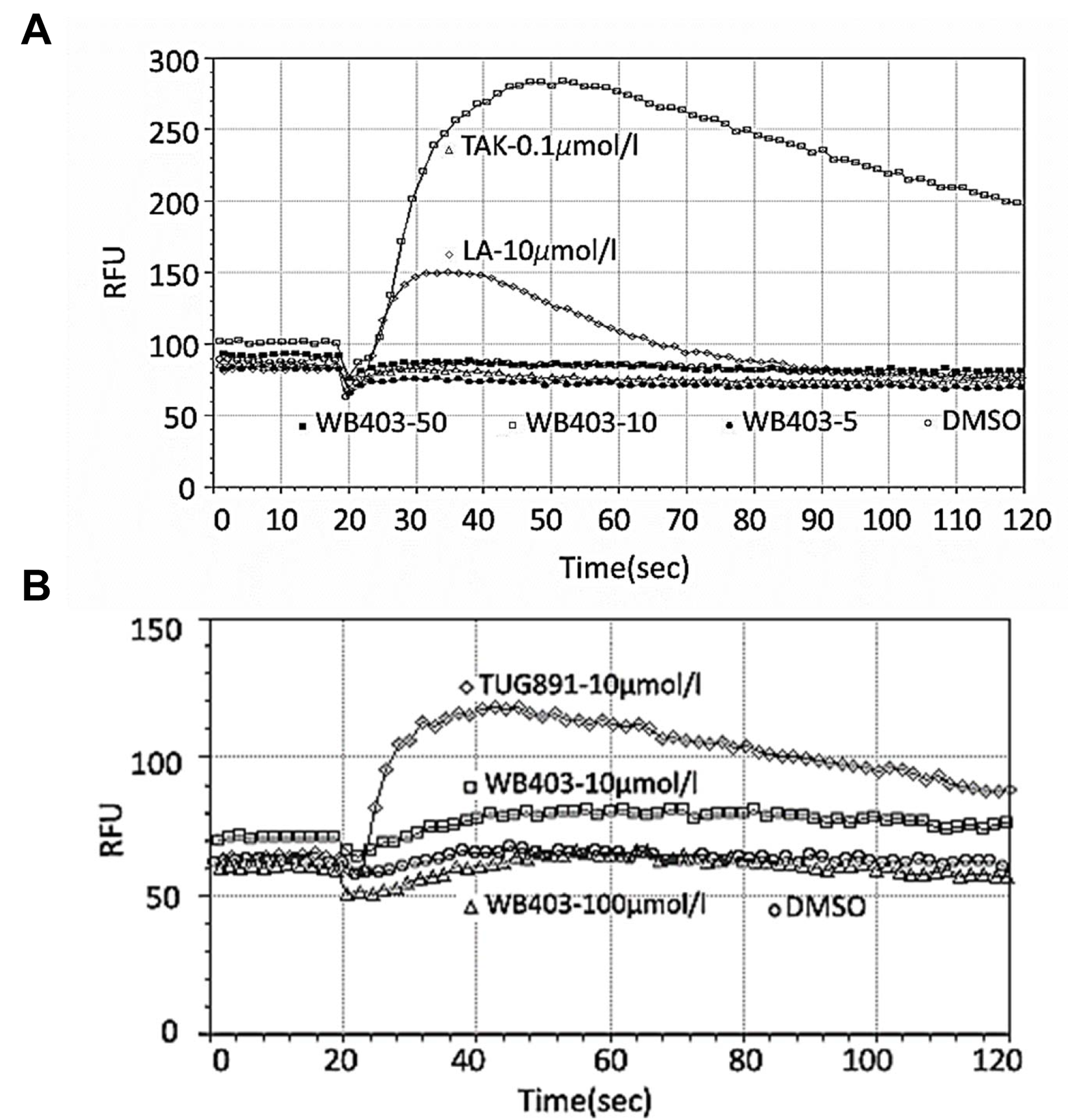

Supplement: S5 Fig — (A) In 293-GPR40 stable cells, WB403 at the concentration range of 5–50 μmol/l did not exhibit significant effect on calcium mobilization. (B) In 293 cells transient transfected with human GPR120 expression vector, WB403 at the concentration range of 10–100 μmol/l did not exhibit significant effect on calcium mobilization. (TIF) [file pone.0134051.s006.tif]
